# A smart automatic control and monitoring system for environmental control in poultry houses integrated with earlier warning system

**DOI:** 10.1038/s41598-025-17074-2

**Published:** 2025-08-27

**Authors:** Abdallah Elshawadfy Elwakeel

**Affiliations:** Agricultural Engineering Department, Faculty of Agriculture and Natural Resources, Aswan University, Aswan, 81528 Egypt

**Keywords:** Global warming, Monitoring system, Poultry houses, Greenhouse gases, Sustainable agriculture, Engineering, Physics

## Abstract

Monitoring key environmental parameters—such as temperature, humidity, ammonia (NH_3_), and methane (CH_4_)—is critical for optimizing poultry health, improving productivity, and mitigating greenhouse gas (GHG) emissions. These variables not only influence poultry well-being and performance but also contribute significantly to environmental pollution, underscoring the need for accurate, continuous, and cost-effective monitoring solutions. The integration of Internet of Things (IoT) technologies offers a transformative approach in agribusiness, enabling real-time data acquisition, automated control, and enhanced connectivity for environmental management in poultry houses. This study introduces a low-cost, automated monitoring and control unit (AMCU) designed for small-scale poultry operations. The AMCU is IoT-based, employing Global System for Mobile Communications (GSM) for communication. The performance of the developed AMCU was evaluated and calibrated under controlled laboratory conditions at the Agricultural Engineering Department, Aswan University, during August 2023, where ambient temperatures ranged between 40 and 42 °C. Each test was replicated three times to ensure consistency and reliability. The results demonstrated a strong correlation (*r* > 0.96) between the AMCU sensor readings and those obtained from certified reference devices, confirming the system’s accuracy in measuring temperature, humidity, ammonia, and methane. The economic analysis revealed that the complete system, including the early warning feature, was constructed at a total cost of only USD 76, with the core measuring unit costing USD 37.5. In contrast, the combined cost of the commercial reference devices was approximately USD 321, indicating that the AMCU achieved comparable functionality at just 11.68% of the commercial cost. The findings suggest that the AMCU is a promising, scalable solution for environmental monitoring in poultry farming. Its future deployment in real-world poultry houses could significantly reduce GHG emissions and promote more sustainable agricultural practices.

## Introduction

A crucial aspect in the advancement of human civilization is in the domain of agriculture^1,2^. Agricultural production or rural activities can involve the emission of unpleasant gases, malodors, or most commonly, greenhouse gases. In any case, the control and monitoring of such emissions in rural, unattended, and remote locations represent an issue in need of addressing^3^. With the global population on the rise, there is a growing need for poultry products. One potential approach to address this requirement is to enhance productivity by expanding housing capacity and effectively managing a larger number of birds. Nevertheless, the combination of this practice, personnel shortages, and the implementation of stricter biosecurity measures will pose growing challenges for producers in monitoring the poultry house environment^4,5^. The United States is the greatest producer of poultry meat globally, accounting for 17% of total output, succeeded by China and Brazil. China is the predominant egg producer globally, accounting for 38% of total output, followed by the United States and India, each contributing 7%. Asia constitutes the predominant egg-producing region, accounting for about 64% of global production. In response to increasing demand, global poultry meat output escalated from 9 to 133 million tonnes between 1961 and 2020, while egg production surged from 15 to 93 million tonnes. In 2020, poultry meat constituted about 40% of worldwide meat output. Over the past thirty years, global egg output has risen by 150%. A significant portion of this rise has occurred in Asia, where production has nearly quadrupled. Approximately 80% of rural households in developing nations engage in poultry farming^6^. In October 2024, the U.S. Department of Agriculture (USDA) projected that global poultry production will increase by 2% in 2025, reaching a record 104.9 million tons, with the most significant growth anticipated in China, the U.S., Türkiye, and Brazil. The industry approaches 2025 in response to the increasing demand for poultry meat. In September 2024, Rabobank projected that consumption in 2024 would increase by 2.5-3%, more than double the growth observed in 2023, with demand significantly accelerating in Europe, China, the Middle East, and Southeast Asia^7^.

The poultry house’s atmosphere is a critical element for productivity that may be monitored and enhanced^8^. Environmental factors that affect the surroundings include temperature, ventilation rate, air velocity, humidity, litter quality, and gas concentrations, such as NH_3_, carbon dioxide (CO_2_), and CH_4_^9,10^. Pereira et al.^11^ emphasized the importance of continuous examining of environmental parameters, including humidity, temperature, NH_3_, and luminosity, due to their direct influence on poultry performance and welfare. Monitoring and controlling the raising environment are a prominent subject in poultry research. The Temperature-Humidity Index (THI) is used to measure the degree of heat stress experienced by animals^12,13^. Heat stress leads to decreased body weight in birds and results in a decline in both the quantity of eggs produced and the quality of the eggshells^14,15^. Humidity, when combined with high temperature, facilitates the proliferation of bacteria, leading to the decomposition of organic matter and the production of NH_3_^16,17^. The temperature should be maintained within a regulated range of 26 to 34 °C, while the humidity should be kept between 50 and 70%^18^. Lower levels of humidity can cause dusty dwelling and increase heating bills, while greater levels of relative humidity can result in moist litter and high amounts of NH_3_^19^. Poultry houses produce noxious gases, such as NH_3_, through the metabolic processes of animals and the decomposition of animal waste. The predominant harmful gas generated in poultry barns is NH_3_ together with CH_4_^13,20^. There is a lack of an adequate system for the management of NH_3_ and CH_4_ gases. Where they pose a danger to the well-being of poultry. Therefore, the presence of an excessive amount of NH_3_ gas in the poultry environment leads to the occurrence of diseases. Regulating the presence of NH_3_ gas in farms is crucial^21^.

At present, there are various commercially available technologies used to monitor environmental factors in poultry production systems. However, ongoing research is focused on developing more sophisticated systems for environmental monitoring and management^10,22^. Studies have demonstrated that multi-sensor systems may successfully evaluate the performance of barn ventilation systems by recording air velocity, differential pressure, and temperature in broiler houses^23^. In a study involving multiple sensor nodes, CO_2_ levels were measured in a controlled chamber. Each sensor showed a linear response to increasing CO_2_ concentrations. However, there were variations in their individual performances, resulting in differences in the measured absolute CO_2_ levels^24^. Jackman et al.^25^ used sensors for temperature, relative humidity, CO₂, and NH_3_, along with bird weight data, to predict broiler weight up to 72 h in advance, with model accuracy rated from good to outstanding. The sensors exhibited a markedly linear response to CO_2_ values, spanning from 500 to 5000 ppm. Nonetheless, the response of each sensor was found to vary, necessitating the separate calibration of each unit. The sensor’s precision varied from 80 to 110 ppm CO_2_, with an anticipated response time of around 5 min to detect a 95% change in concentration. Wan et al.^26^ explored the integration of farm automation systems with field servers on a poultry farm in Taiwan, also investigating the effects of additional environmental sensors, including those for rainfall and infrared radiation. Murad et al.^27^ introduced a monitoring system for poultry farms that relies on wireless sensor networks (WSNs). The system utilizes TelosB motes, which are equipped with commercial sensors capable of accurately measuring temperature and humidity. So-In et al.^28^ introduced an innovative system that combines WSNs with mobile phone integration to monitor environmental behavior. The system promptly notifies the administrator of any unexpected changes. The integration of TelosB and Windows Phone OS was utilized to apply the notion of smart farming to poultry farming over an evaporative cooling system (ECS). Choukidar^29^propose a wireless sensor network integrated with General Packet Radio Service (GPRS) technology for examining and regulating gas, water level, temperature, smoke, and food distribution. A Raspberry Pi is utilized to handle and oversee all data management. Data is transmitted through GPRS, and a webpage is used to keep comprehensive records of the poultry farm, including the current environmental conditions. The study conducted by Lashari et al.^30^ involved the implementation of an IoT-based system for monitoring the environment in poultry farms. This system utilizes a Raspberry Pi and a set of sensors to monitor several parameters like air temperature, air humidity, O_2_ levels, CO_2_ levels, and NH_3_ concentration. The research highlights the importance of maintaining optimal environmental conditions to ensure the health and productivity of poultry. By leveraging IoT technology, the system provides real-time monitoring and data collection, which allows for timely interventions and adjustments to the environment. Fernando et al.^11^ introduced cost-effective software and hardware solutions for monitoring environmental conditions in poultry farms. Comparative studies revealed that the prototype sensors correlated above 0.90 with calibrated equipment and were priced at just 13% of traditional devices. The system effectively measures critical parameters such as temperature, humidity, ammonia levels, and luminosity, which are essential for ensuring animal welfare and optimizing productivity. Suriano and Abulude^3^ monitored gas emissions in agricultural productions through low-cost technologies. Two portable monitoring units developed in the laboratory and based on low-cost gas sensors were used to provide indications about the concentrations of NH_3_, CH_4_, H_2_S, and CO_2_. During this experiment, two monitors were deployed: the first one was placed in the manure storage depot, while the second one was deployed out of the storage site to compare the gas concentrations related to the outdoor environment with the gas emissions coming from the manure. Both devices were wirelessly linked to the Internet, even though the radio signal was weak and unstable in that area. This situation provided us with the opportunity to test a particular protocol based on sending and receiving e-mails containing commands for the remote machines. This experiment proved the effectiveness of the use of low-cost devices for gas emission monitoring in such particular environments.

In addition, the gadgets integrated into smart poultry management systems will be connected to the Internet, enabling the establishment of IoT farm networks. The implementation of IoT technology facilitates connectivity among farm sensors, devices, and equipment, ultimately resulting in the automation of many agricultural processes^5^. Hambali et al.^31^ established an IoT-enabled smart poultry farm in Brunei, where the majority of farms continue to employ manual techniques. Their solution utilizes an Arduino microcontroller along with multiple sensors to regulate temperature, humidity, air quality, illumination, ventilation, and food distribution. Nuyya et al.^32^ conducted a study aimed at developing a control system for a small-scale chicken farm, which was established following the modeling and simulation of the farm environment. Their device, utilizing Arduino technology, was evaluated with approximately 100 quails and intended to control gasses, light, humidity, and temperature. Thus, finding solutions that offer convenient, reliable, and cost-effective monitoring of these variables is essential. In the field of agribusiness, IoT holds great potential. It has been developed as a solution to address the lack of connectivity in this industry by providing sensed data, hence significantly enhancing device connectivity^11^.

The current systems presented in previous studies represent a barrier to small poultry farmers and small-scale farm owners due to the high cost of this technology. In Egypt, we face significant difficulties in obtaining this technology due to its unavailability in local markets. Furthermore, all these systems do not include a warning system for the dangers of some parts of the system malfunctioning. This represents a major obstacle for small farmers who do not have any experience in operating smart control systems. So, based on the above review, the present work is intended to develop and laboratory evaluation of a smart automatic control and monitoring system for environmental control in small-scale poultry houses integrated with earlier warning system based on IoT and GSM. And mitigating GHG emissions in small scale poultry hous es, by regulating temperature, humidity, NH_3_, and CH_4_ emissions. Furthermore, the control and monitoring system is fitted with an early warning system to safeguard the lives of poultry and minimize power losses.

## Materials and methods

### System architecture

**Fig. 1:**
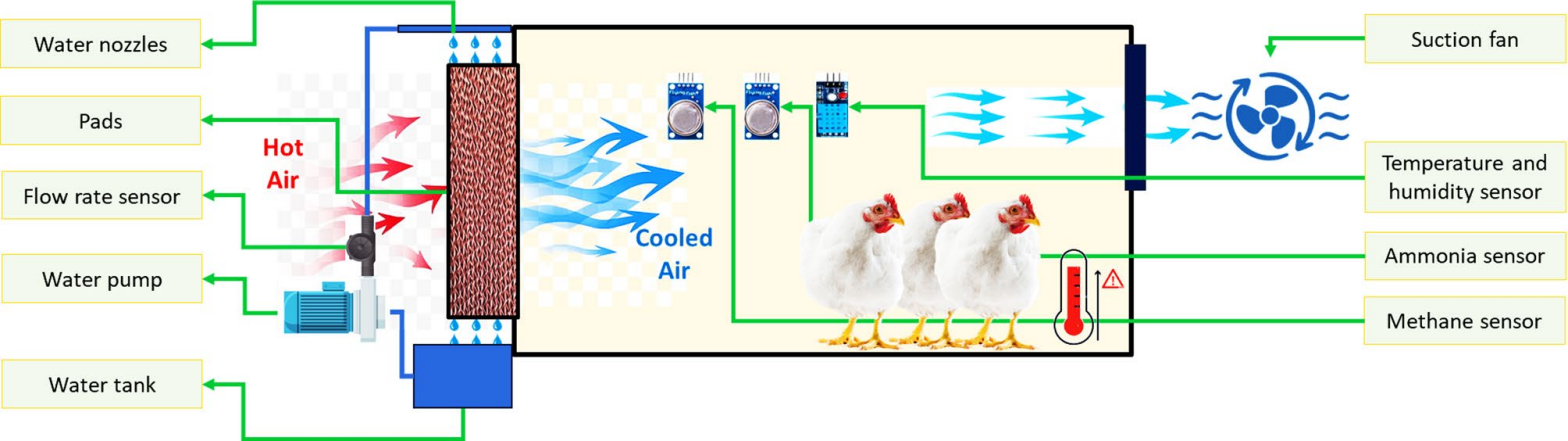
The main components of the developed AMCU.

To achieve the goals of the current study, an automatic monitoring and control unit (AMCU) was created. This unit measures both temperature and humidity inside poultry houses. It is designed to regulate high temperatures by using an ECS that includes a sensor to measure water flow rate. The AMCU also controls the level of humidity to ensure it remains within safe and necessary levels. This is done to prevent the growth of diseases and protect the respiratory system of the poultry from conditions of low or high humidity, which can lead to the spread of dirt, dust, and diseases. In addition, the AMCU was equipped with sensors to continuously monitor the concentrations of NH_3_ and CH_4_ gases inside the poultry houses, which are primarily generated through the fermentation and decomposition of poultry wastes. Elevated levels of these gases are known to adversely affect bird health and can significantly increase mortality rates. To mitigate this risk, the system automatically activates ventilation fans when high concentrations of NH_3_ or CH_4_ are detected, thereby replacing contaminated indoor air with fresh air from outside the poultry houses. Figure 1 explains the main components of an AMCU in poultry houses.

#### Automatic monitoring and control unit (AMCU)

**Fig. 2:**
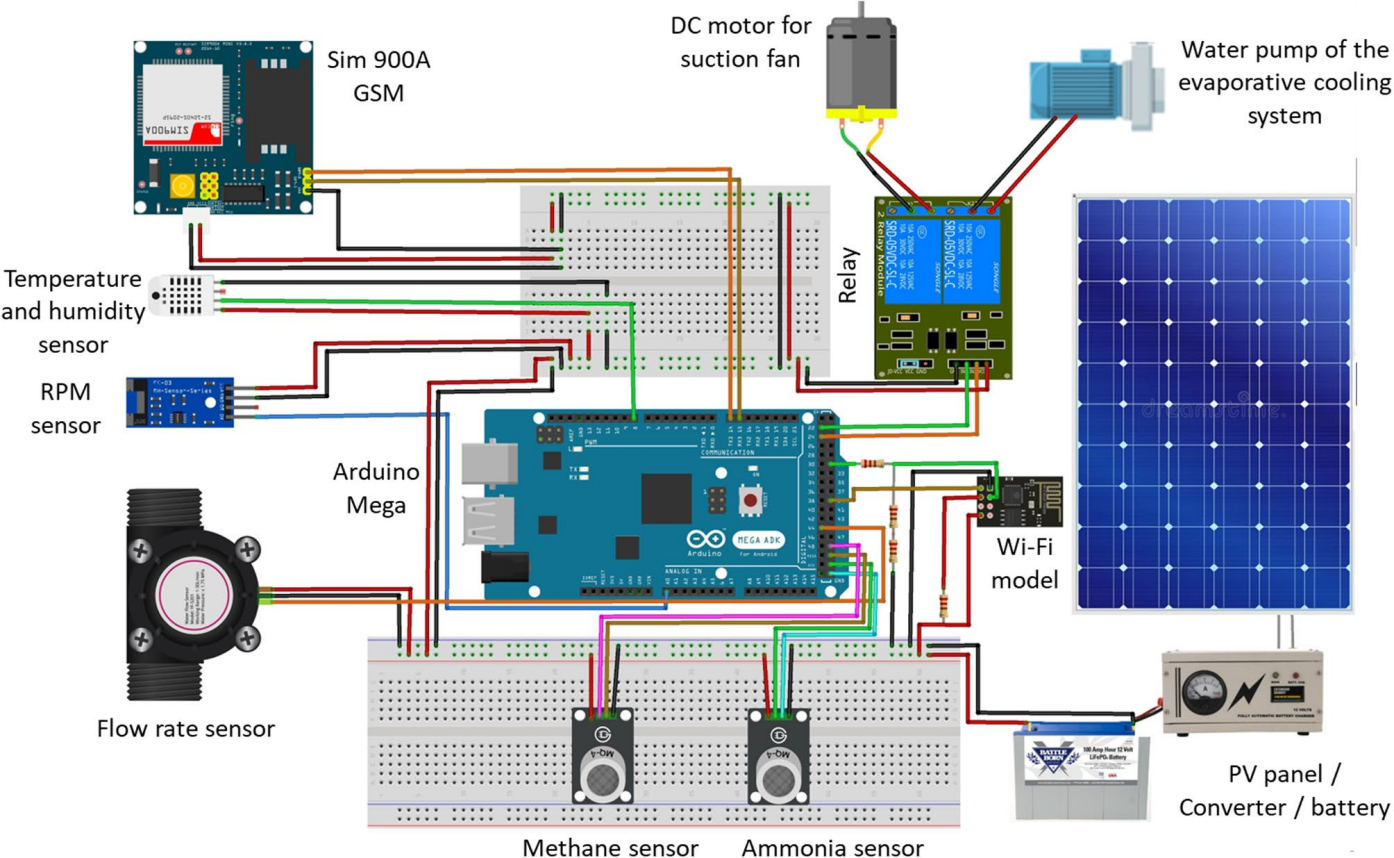
The electronic components with correct and accurate electrical connections for the developed AMCU.

**Fig. 3:**
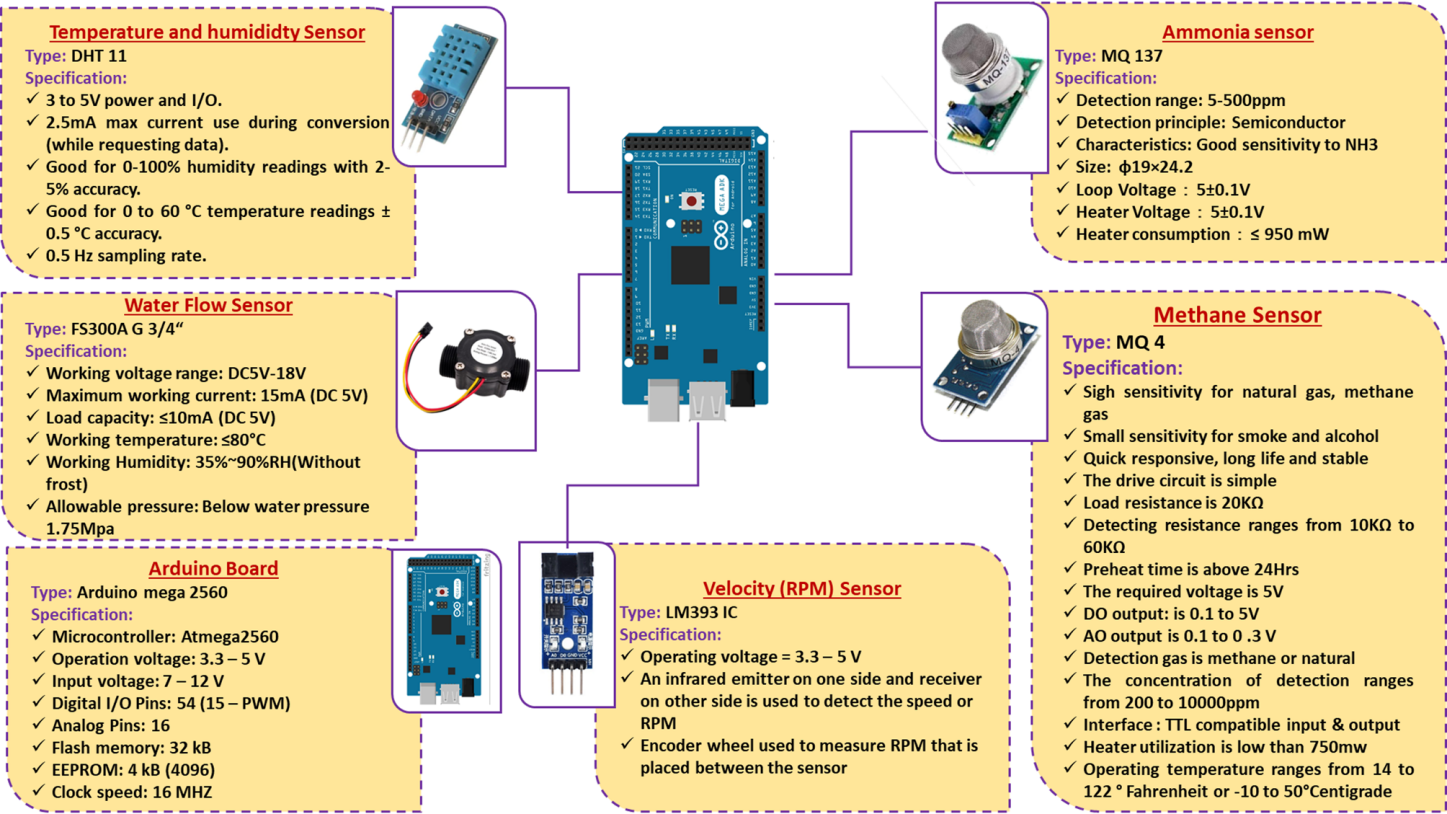
The technical specifications of the Arduino mega board and the different sensors used on the current investigation.

**Fig. 4:**
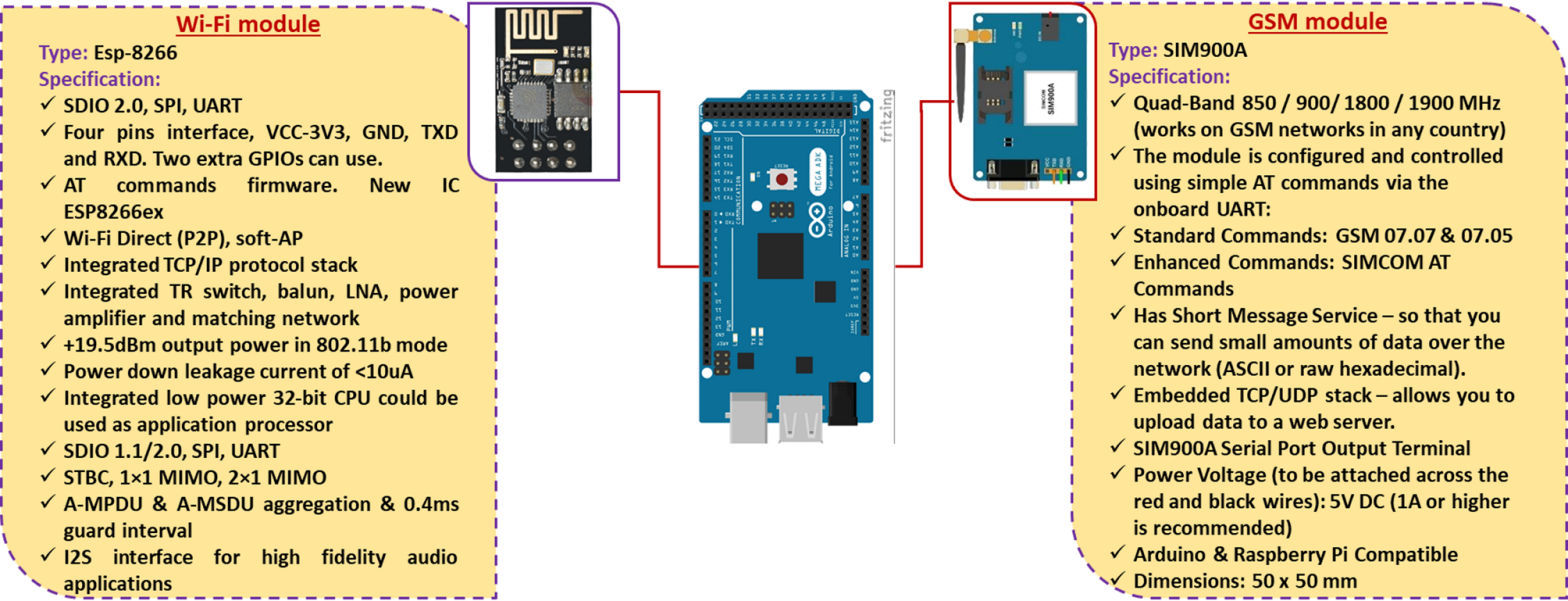
The technical specifications of the GSM module and Wi-Fi module.

Figure 2 illustrates a diagram of a solar-powered ECS for poultry houses. It utilizes solar energy to power the suction (ventilation) fan, water pump of the ECS and other electronic components. Where the system consists of many parts such as: a solar panel for converting sunlight into electricity, which is used to power the suction fan, water pump of the ECS and other electronic components; a battery for storing excess electricity generated by the solar panel, allowing the AMCU to operate even when sunlight is not available (night); a suction fan to draw air into the bottom of the poultry house and passes it over the wet cooling pads; water pump for lifting the water from reservoir to water nozzles then to pads; an Arduino mega board (AMB) for receiving readings from various sensors, then processes the data and finally, makes the appropriate decision based on the operating algorithms, an NH_3_ and CH_4_ sensors to measure the percentage of NH_3_ and CH_4_ gases in the atmosphere of the poultry houses and send the readings to the Arduino mega board; a speed (RPM) sensor to count and estimate the speed of the suction fan and send the readings to the Arduino mega board; an water flowrate sensor to measure the water flowrate on the ECS and send the readings to the Arduino mega board; a Wi-Fi module To send readers to the monitoring system from the operating Arduino, periodically throughout the day and night; a GSM module to send warning text messages (SMS) from the Arduino mega board to the operator if a technical error occurs that could lead to production losses if it is not resolved quickly. Figure 3 shows the technical specifications of the Arduino mega board, and the different sensors used on the current investigation. Figure 4 shows the technical specifications of the GSM module and Wi-Fi module. Table 1 shows the models and functions of different hardware configurations.

**Table 1:** Models and functions of different hardware configurations.

No.	Hardware components	Model	Connected pins with Arduino mega board	Functionality
1.	Arduino board	Mega 2560	–	Controlled all parts of the developed system.
2.	GSM module	SIM900A	14–15	Used for sending a warning message to the operator in case of system failure.
3.	Wi-Fi module	ESP-8266	31–39	Used for sending the measured data via internet to the user interface.
4.	RPM sensor	LM 393 IC	A0	Used to measure the speed of the ventilation fan and is pivotal in the early warning system in case the ventilation fan stops due to damage or power outage in the event of high temperatures.
5.	Methane sensor	MQ4	47–49	Used for measuring the methane concentration of the air inside the poultry houses, it was necessary for controlling air ventilation system according to the preset points.
6.	Ammonia sensor	MQ137	51–53	Used for measuring the ammonia concentration of the air inside the poultry houses, it was necessary for controlling air ventilation system according to the preset points.
7.	Water flow rate sensor	FS 300 AG 3/4 ‘’	44	Used to measure the water flow rate to the ECS and is pivotal in the early warning system in case water does not reach the ECS in the event of high temperatures.
8.	Temperature sensor	DHT-11	8	Used for measuring the air temperature inside the poultry houses, it was necessary for controlling both an ECS and an air ventilation system according to the preset points.
9.	Humidity sensor	DHT-11	8	Used for measuring the relative humidity inside the poultry houses, it was necessary for controlling the ECS and adjusting the relative humidity according to the present points.

#### Evaporative cooling systems (ECSs)

**Fig. 5:**
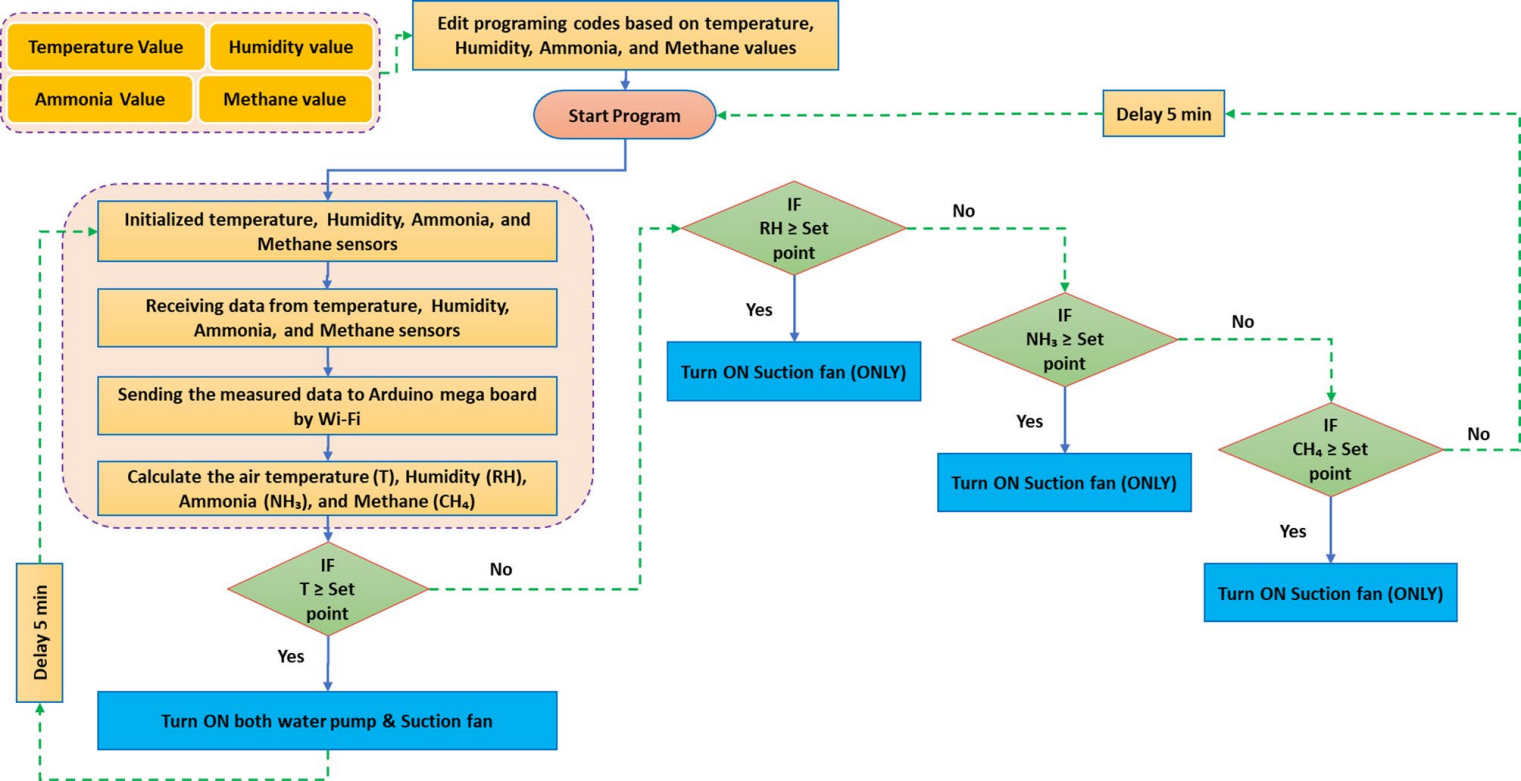
The main components of the developed AMCU.

**Fig. 6:**
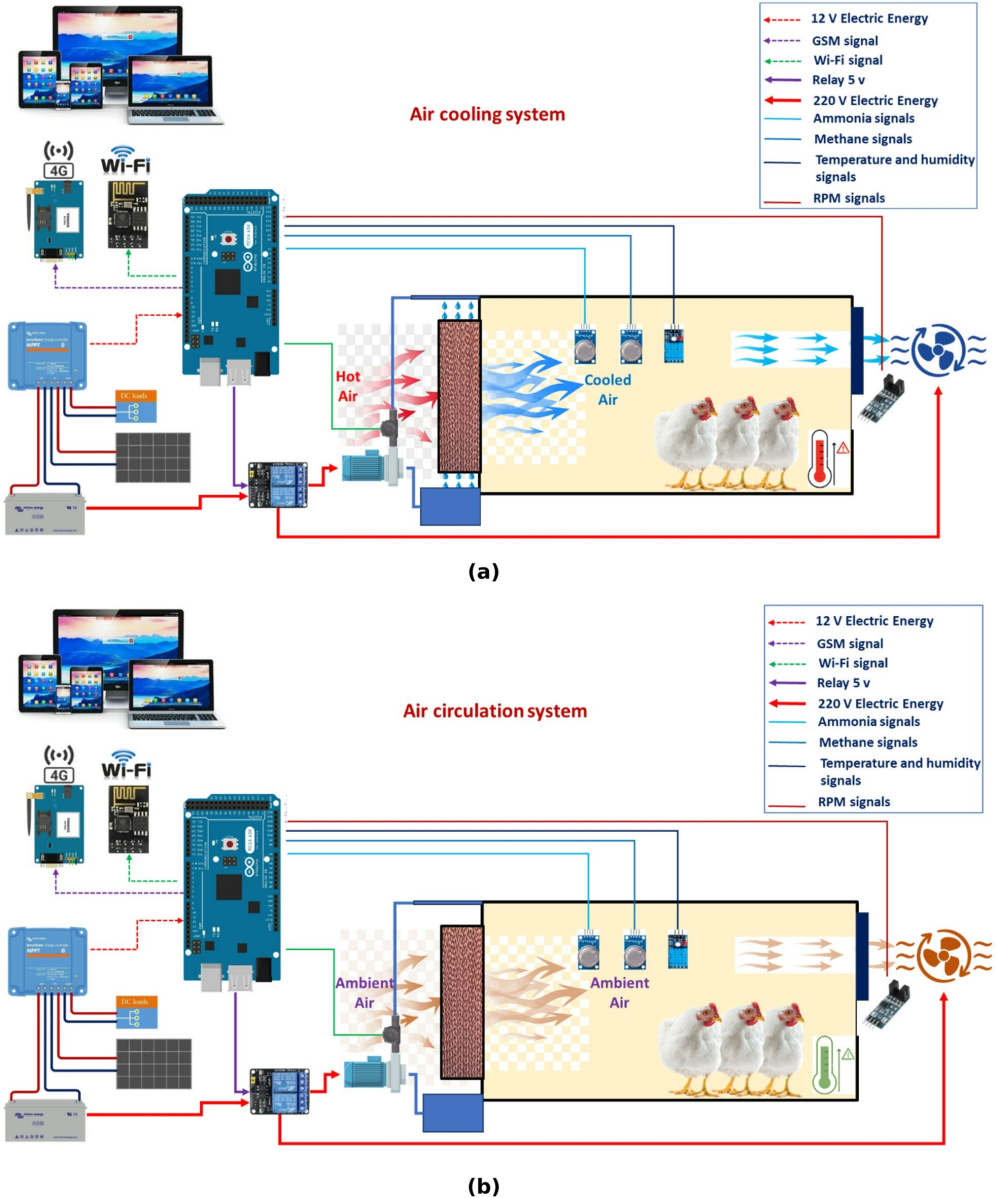
Main components of the developed AMCU, whereas (**a**) represents the air-cooling system when the air temperature inside the poultry house is higher than the surrounding environment, while (**b**) represents the air circulation system when the air temperature inside the poultry house is in equilibrium with the surrounding environment.

Figures 5 and 6 show a diagram of ECS specifically designed for poultry houses. ECS is a natural process that utilizes water evaporation to cool the air. This system effectively reduces the temperature within the poultry houses, providing a more comfortable environment for the poultry. The ECS is an intelligent indoor equipment system designed to regulate and optimize the atmosphere for animal breeding sectors worldwide, with a particular focus on the food business, specifically poultry farming, in agricultural countries like Thailand^33^. The system’s environmental monitoring and control are crucial due to its autonomy from natural factors. Typically, ECSs utilize a water-cooling fan-pad along with a fan-controller that may include an optional vent box and curtain controller. These features are primarily utilized to modify the air volume flow in order to regulate temperature and humidity levels in various conditions^34^. The ECS consists of several components such as: (1) Water pump: The water pump maintains constant water pressure, providing the necessary force to deliver water to the cooling pads. Adequate water pressure ensures that the pads receive a sufficient amount of water for effective cooling. The performance of the water pump directly impacts the effectiveness of the ECS. A malfunctioning or inefficient water pump can lead to uneven water distribution, reduced cooling capacity, and increased water consumption. Consequently, it is essential to maintain the water pump regularly and ensure its proper operation for optimal system performance; (2) Water nozzles: These nozzles spray water onto a series of cooling pads, which are typically constructed from cellulose or fiberglass; (3) Suction fan: a powerful fan draws air into the bottom of the poultry house, passing it over the wet cooling pads; (4) Water tank to store the water that is used to wet the cooling pads. The tank should be sized correctly to accommodate the system’s requirements and the number of poultry in the poultry house.

The ECS in this barn was chosen to: (1) Reduce heat stress in poultry: By lowering the temperature in the poultry house, evaporative cooling can significantly reduce heat stress in poultry, improving their overall health and well-being^35,36^; (2) Maintain optimal humidity levels: ECSs effectively control humidity levels within the poultry house, preventing excessive moisture buildup that can lead to respiratory problems and other health issues for the poultry^37,38^; (3) Reduce GHG emissions: By removing heat from the poultry house, evaporative cooling can help to reduce the production of GHG emissions^39–41^which are harmful gases produced by poultry waste, where in a tunnel-ventilated swine building, fitted with evaporative cooling pads across the entire end of the building 1.2 m upwind from the exhaust fans, researchers measured a 33 and 50% reduction in NH_3_ levels and a 20 and 60% reduction in total dust at high and low ventilation rates, respectively^42,43^; (4) Improved egg production: Studies have shown that providing poultry with a comfortable environment, including adequate cooling, can lead to increased egg production. Where Gates et al.^44^ reported that, heat stress in both mechanically and naturally ventilated egg production facilities is a problem for the egg industry. Various means of providing supplemental cooling to hens in facilities are available, including evaporative cooling from either pads or from misting or fogging systems. Furthermore, ECSs exhibit higher energy efficiency in comparison to conventional air conditioning systems, rendering them a financially viable choice for cooling poultry houses. This type of system is particularly well-suited for warmer climates where poultry are susceptible to heat stress. Also, solar-powered ECSs are cost-effective and environmentally friendly.

### Operating algorithm

#### Operating algorithm of the AMCU

Figure 5 depicts the operational algorithm used to compute the values of air temperature, humidity, NH_3_, and CH_4_. The operational algorithm commences by modifying the programming codes of the control system according to the specified values for air temperature, humidity, NH_3_, and CH_4_. Where the extent of operation is determined for each of the previous sensors, which includes the lowest value and the highest value for each of the previous gases, as this is the permitted term and the safe limits of operation. After that, all sensors and Arduino mega board are initialized for the operation and gases readings to measure their percentage in the atmosphere of poultry houses. Then, the AMB reads the analog values from the air temperature, humidity, NH_3_, and CH_4_ and converts them to digital values. Then, digital values are used to calculate the air temperature value and the percentage of humidity, NH_3_, and CH_4_ in the atmosphere of poultry houses.

If the air temperature was equal to or higher than the set point, the Arduino mega board will give the water pump operation signal (ECS) as well as a signal to operate the air suction fan, to replace hot air inside the poultry house in a cold, through the ECS (Fig. 6a). On another hand, if the percentage of humidity or NH_3_ or CH_4_ were equal to or higher than the set point, the Arduino mega board will give a signal to operate the air suction fan (only), to renew the air inside the poultry house and reduce the percentage of previous gases (Fig. 6b).

#### Decision flowchart of the developed AMCU

Any electronic control system is susceptible to potential malfunctions arising from the failure of certain automatic components. In the present system, for example, the cooling process relies on both water and air to maintain suitable temperature levels inside the barn. However, if a malfunction occurs in the water supply system—such as an obstruction preventing water from reaching the cooling pads, a reduction in cooling water availability, or the complete absence of water in the storage tank connected to the ECS—the system is designed to automatically detect the fault.

This detection is based on monitoring the water flow rate from the cooling pump. If the measured flow rate is greater than zero, it confirms that water is being delivered properly and the system is operating efficiently. Conversely, if the flow rate equals zero, it indicates a malfunction in the water circulation system. In such cases, the ECS immediately sends an alert via the GSM module to the operator, prompting a rapid inspection and corrective action to restore normal operation.

**Fig. 7:**
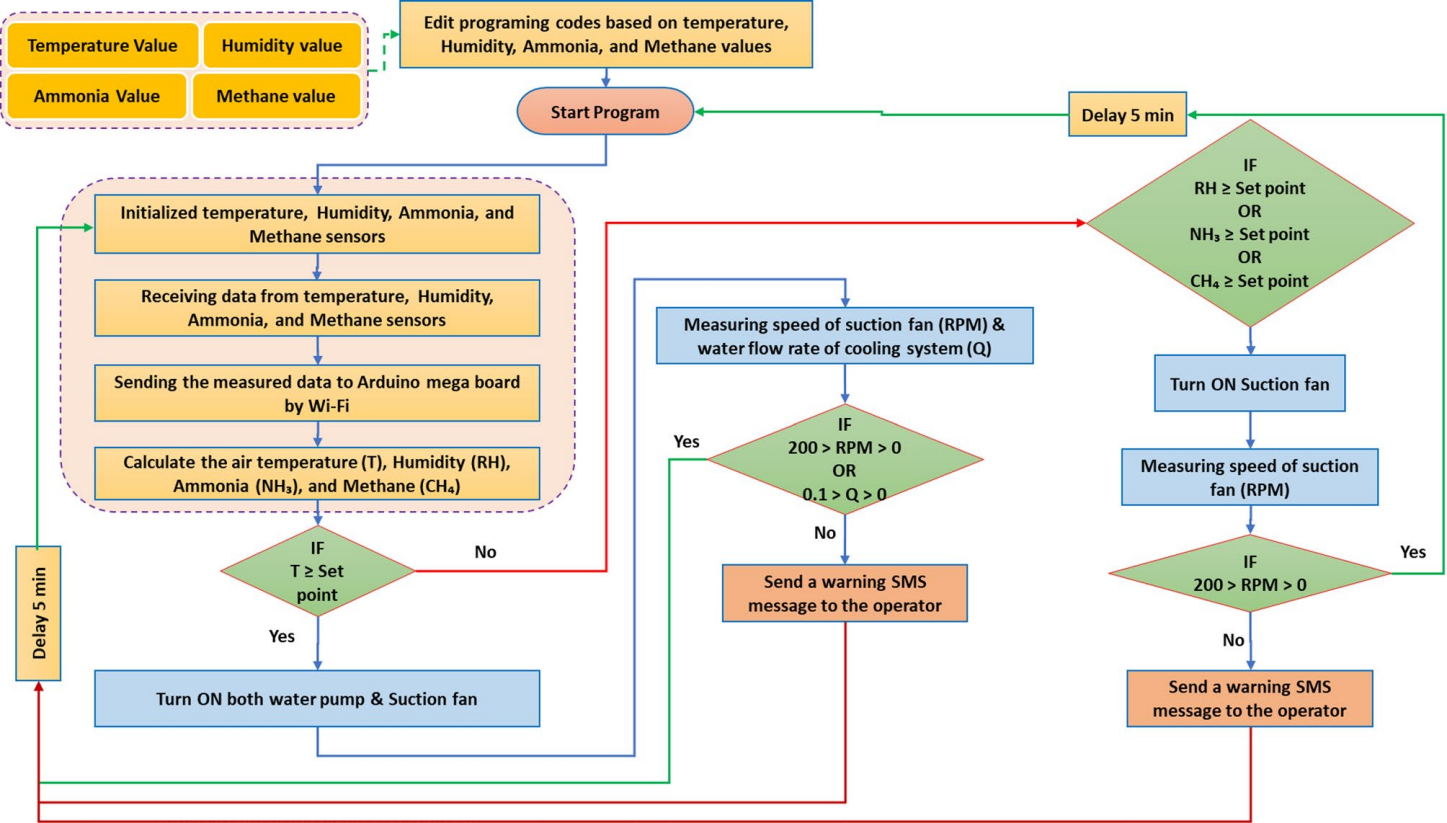
Decision flowchart of the developed AMCU.

To further enhance reliability, an early warning circuit (Fig. 7) was integrated to detect malfunctions in the air suction fan. A speed sensor was installed to monitor the fan’s rotational speed. Under normal conditions, when the system detects elevated levels of temperature, humidity, or NH_3_, it automatically activates the suction fan to replace contaminated barn air with fresh air. The speed sensor then confirms fan operation. If the fan speed remains zero despite activation, the system recognizes a failure and, as with the cooling pump, sends an automatic text message through the GSM network to notify the operator for immediate intervention and system restart.

#### Operating algorithm of the communication systems

**Fig. 8:**
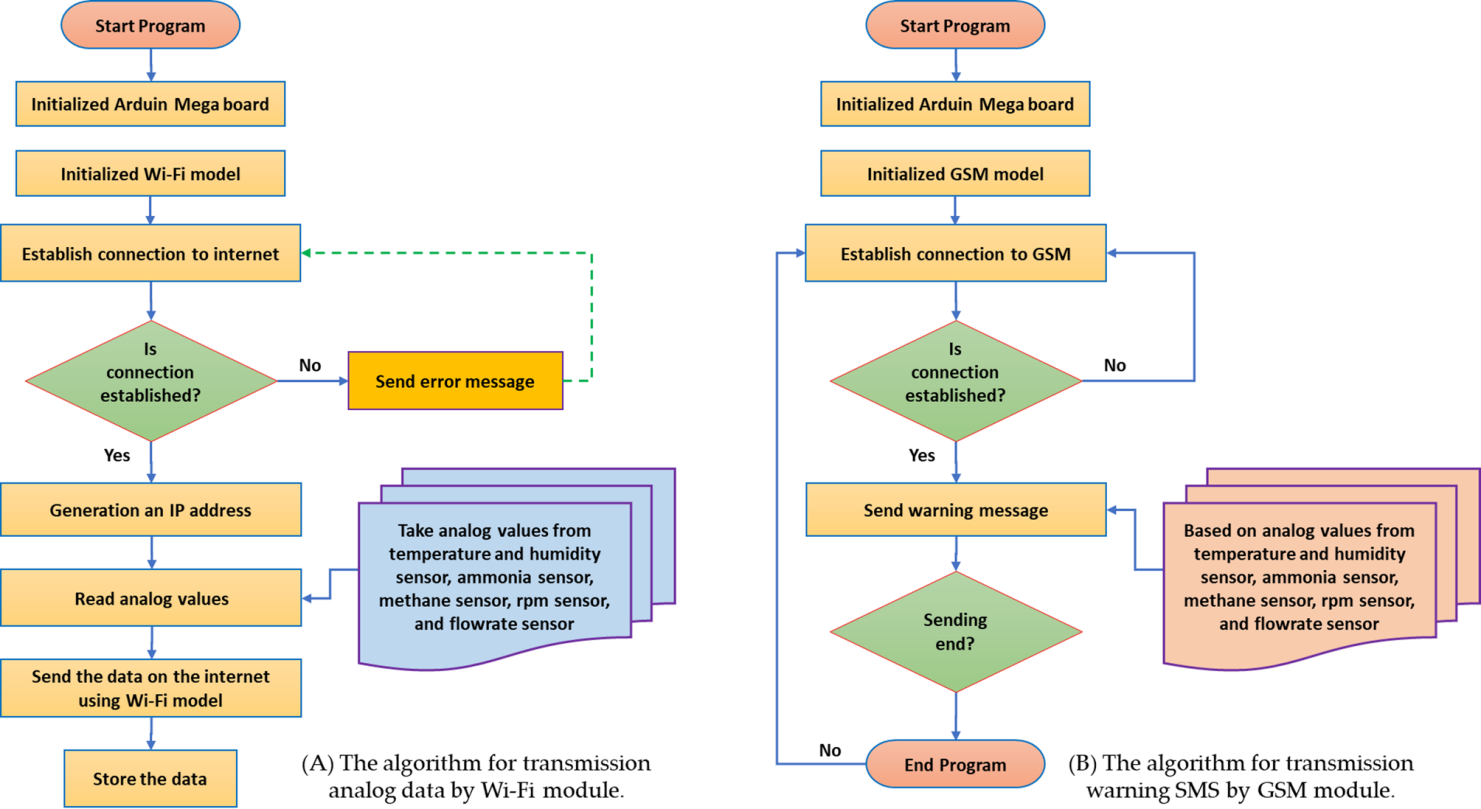
Operating algorithm of communication systems.

Figure 8 shows the operating algorithm for transmission analog data by Wi-Fi, which collects data from different sensors then sends it to the user interface (smart phone or laptop) via internet using a Wi-Fi unit. It starts with initializing the AMB and the Wi-Fi unit. Then, it tries to establish a connection to the internet. If the connection is successful, the program generates an IP address and takes analog values from the temperature sensor, pH sensor, and ultrasonic sensor. It then reads the analog values and sends them to the internet using the Wi-Fi unit.

In the current study to reduce errors, two different types of communications were used. The first type, Wi-fi was used to send the readers of the readers of the freshness of the heat, the relative humidity, the percentage of NH_3_ and the percentage of CH_4_ gas in the amber atmosphere periodically every hour to the operator through the mobile application. The second type, GSM, was used to send a warning text message in the event of refrigeration of the cooling or air suction fan for doing the specific function of each of them and thus the possibility of avoiding the problem that can negatively affect production.

## Results and discussion

**Fig. 9:**
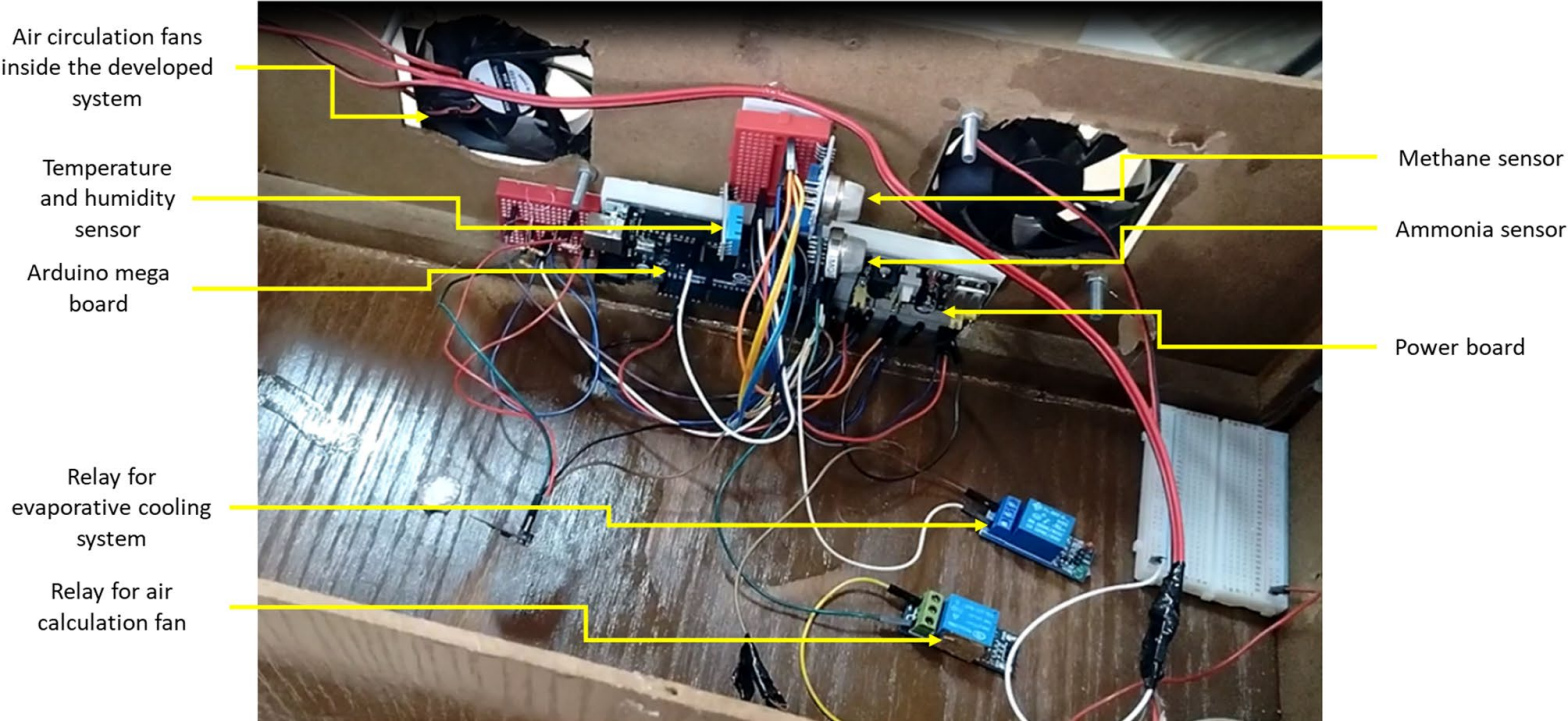
Prototype showing the main components of the developed AMCU during laboratory tests.

The regression analyses were performed using Microsoft Excel 2019, which allowed both curve fitting and statistical validation of the sensor outputs. Data were obtained under controlled laboratory conditions at the Agricultural and Engineering Department, Aswan University, during August 2023. Measurements were recorded every 10 min over a 30-day period, with each experiment repeated at least three times to ensure consistency. Average values from repeated trials were used for regression modeling and curve plotting. This ensured that the derived regression equations were based on stable, representative datasets, while minimizing the influence of random fluctuations. Figure 9 shows a prototype showing the main components of the developed AMCU during laboratory tests.

### Temperature and humidity sensor

**Fig. 10:**
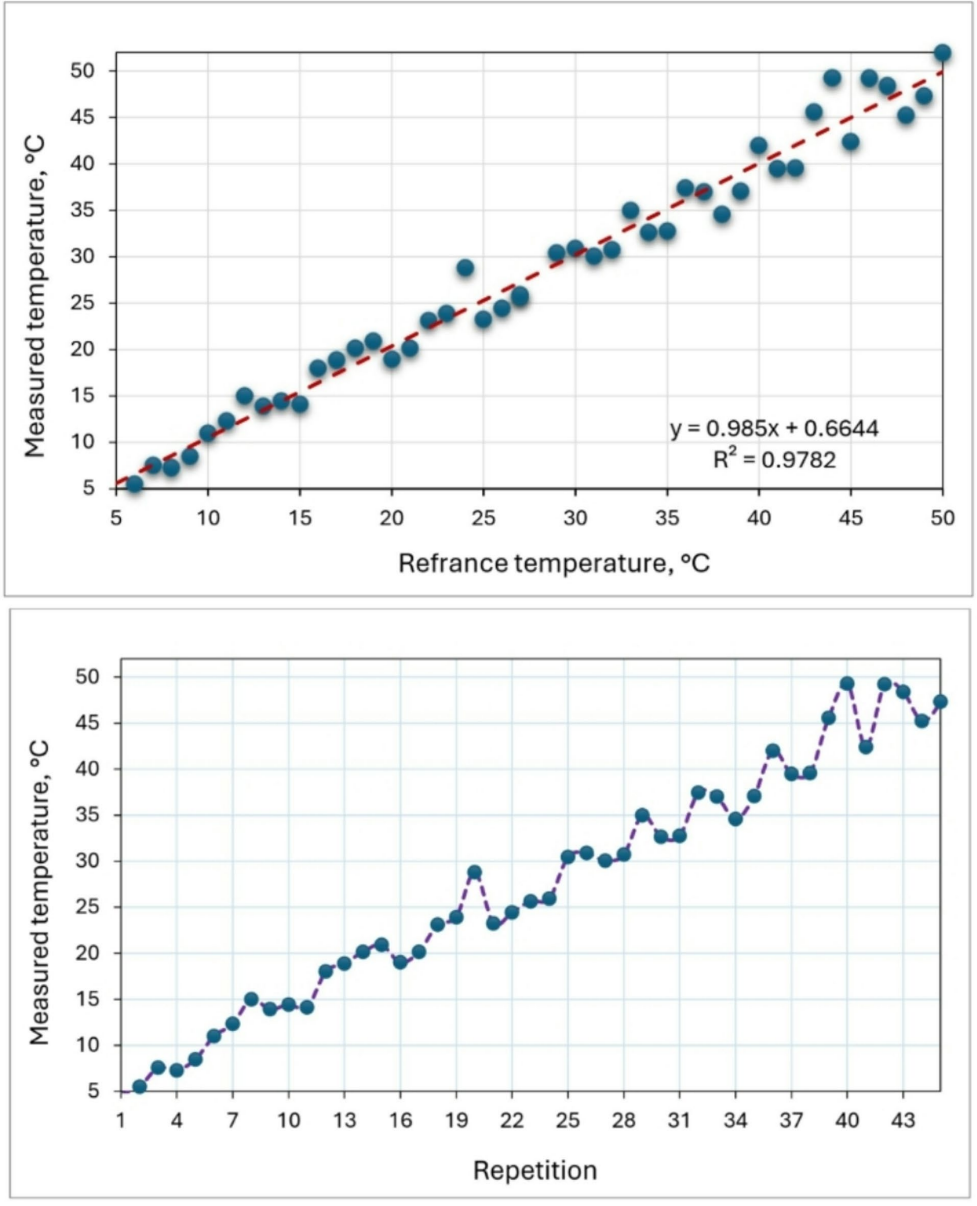
Calibration of the air temperature sensor by reference measuring device.

**Fig. 11:**
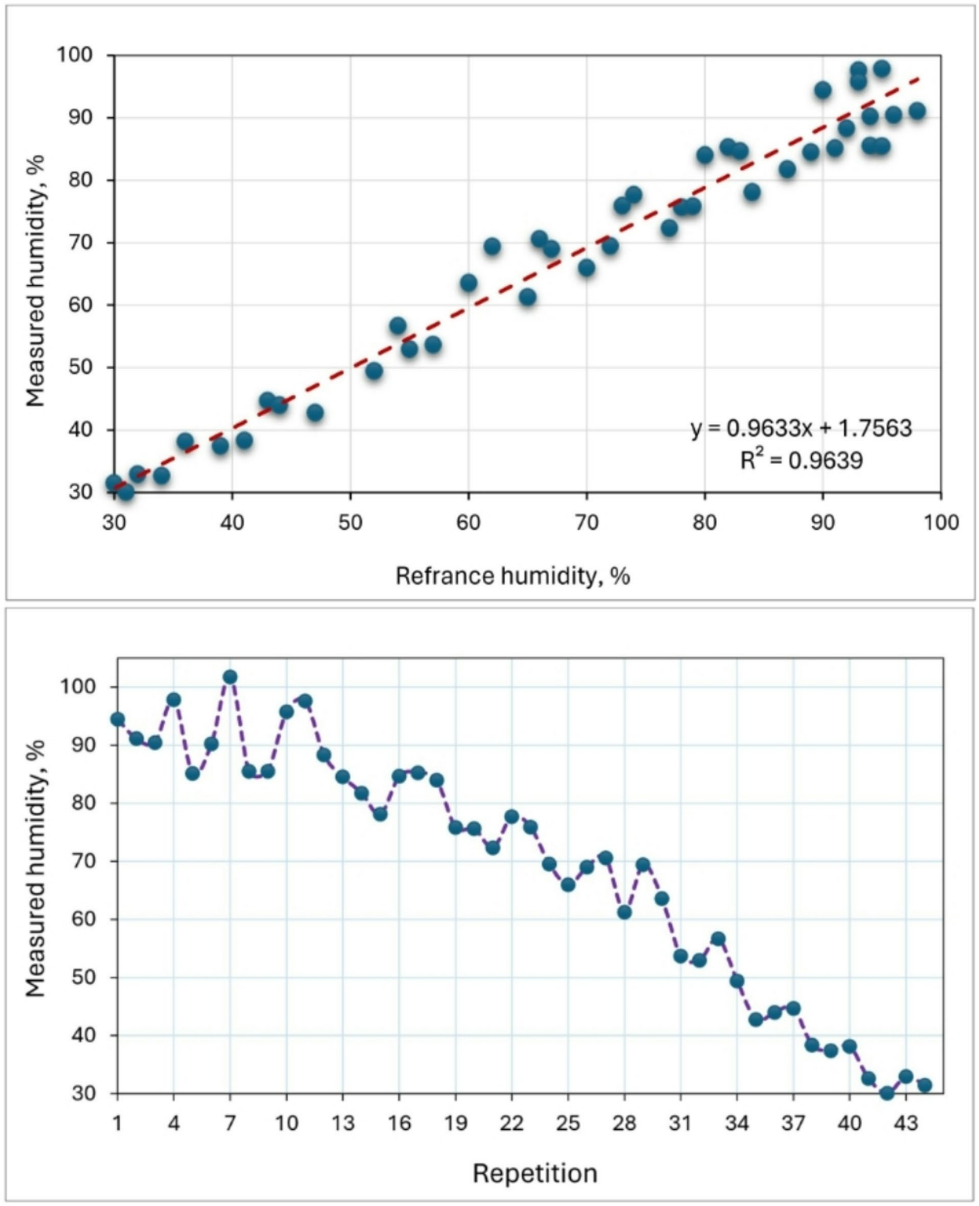
Calibration of the relative humidity measured sensor by reference measuring device.

Figures 10 and 11 show the calibration of the air temperature measured by the developed measuring system and reference measuring device. The calibration was applied to temperature and humidity sensor (model: DHT-11). The reference temperature was recorded using a digital temperature and humidity meter (model: UT333s). The temperature sensor was placed in a closed container containing ice, and the temperature gradually increased by an external heat source. During this period, the temperature was measured periodically by the temperature sensor and by the standard device. The humidity sensor was also exposed to airflows of varying temperatures. High-temperature air had low humidity, while low-temperature air had high humidity. Humidity was measured using both the humidity sensor and a standard measuring device. The temperature sensor was calibrated at different temperature levels ranging between 5 and 50 ᵒC. The recorded data were then plotted on the x-axis, with the temperature and humidity readings from the DHT-11 sensor plotted on the y-axis for each calibration state. Validation of the DHT-11 sensor measurements against the reference data showed high correlations, with R^2^ values of 0.9782 for air temperature and 0.9639 for air humidity. This indicates a strong alignment between the reference measurements and those taken by the DHT-11 sensor integrated into the measuring and control circuit. The DHT-11 sensor demonstrated excellent performance across various temperature and humidity levels, with linear regressions of y = 0.985x for air temperature and y = 0.9633x for air humidity, closely matching the 1:1 line. Calibration of the DHT-11 sensor with the UT333s resulted in a temperature measurement accuracy of 99.8% as reported by^45^. Elwakeel et al.^46^ used DHT-11 sensor for measuring dry air temperature and relative humidity of ambient air and inside solar collector and drying room.

### Water flowrate sensor

**Fig. 12:**
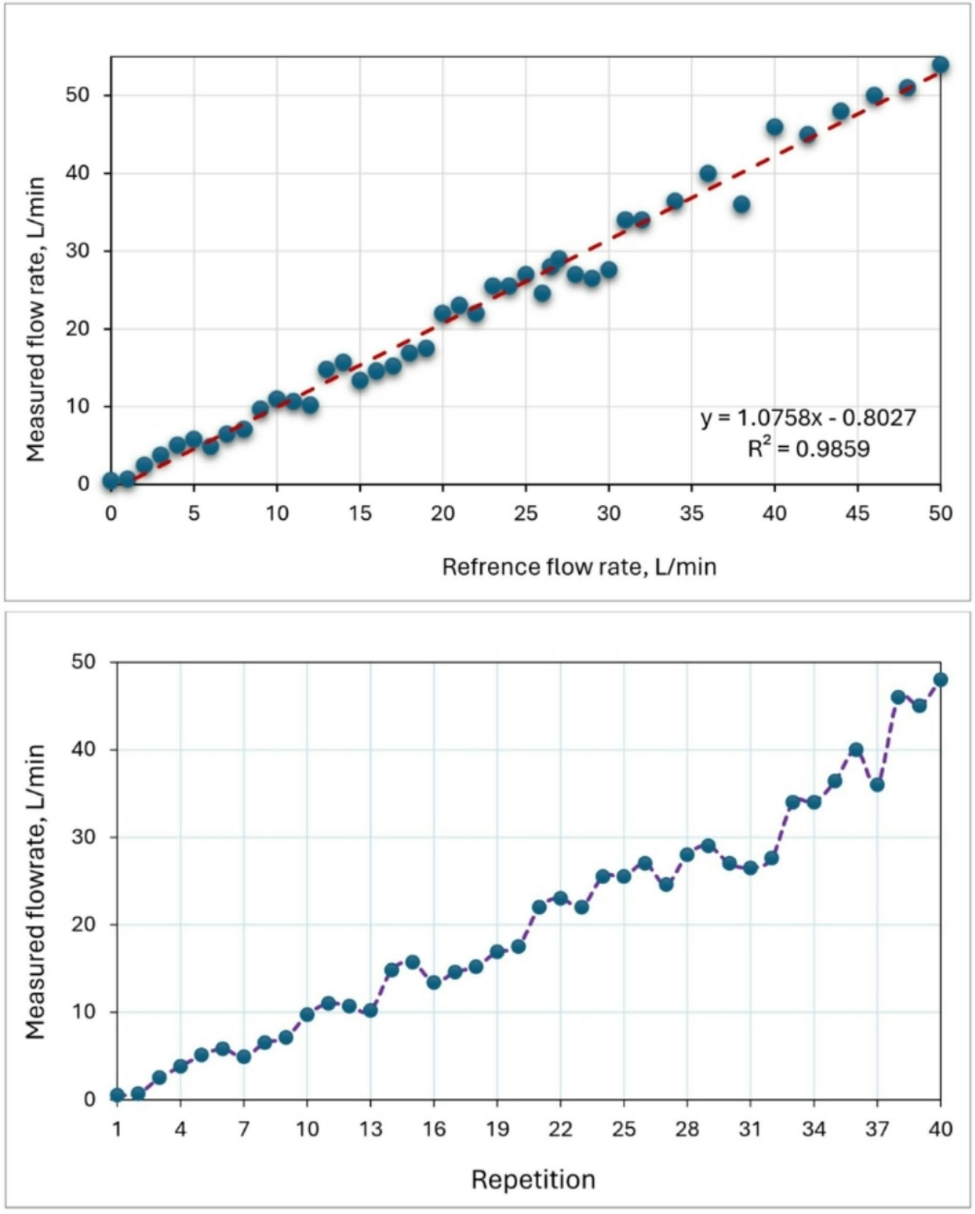
Calibration of the water flowrate sensor by reference measuring device.

The water flowrate sensor [model: FS300A G 3/4] was used in the current study for measuring water flowrate for the ECS. Figure 12 shows the calibration of the water flowrate sensor by reference measuring device; the water flow rate sensor was calibrated in a range of 0–50 L/min. The measured water flowrate detected by the FS300A sensors has been confirmed with the reference flowrate measured by the traditional method [estimating time required to fall a certain volume] with high R^2^ value of 0.9859. This implies that the linear model can account for 98.59% of the variability in the data. Easa et al.^47^ employed the FS300A sensor to quantify the water output of a solar desalination system that included a high-speed rotary humidifier. Kaltakkiran^48^ investigated the impact of coolant strategy on the immediate energy balance during the warm-up phase in a spark ignition engine. In this study, FS300A flowmeters with a precision of 3% were employed to measure the coolant flow rate in the experimental setup.

### Fan speed sensor

**Fig. 13:**
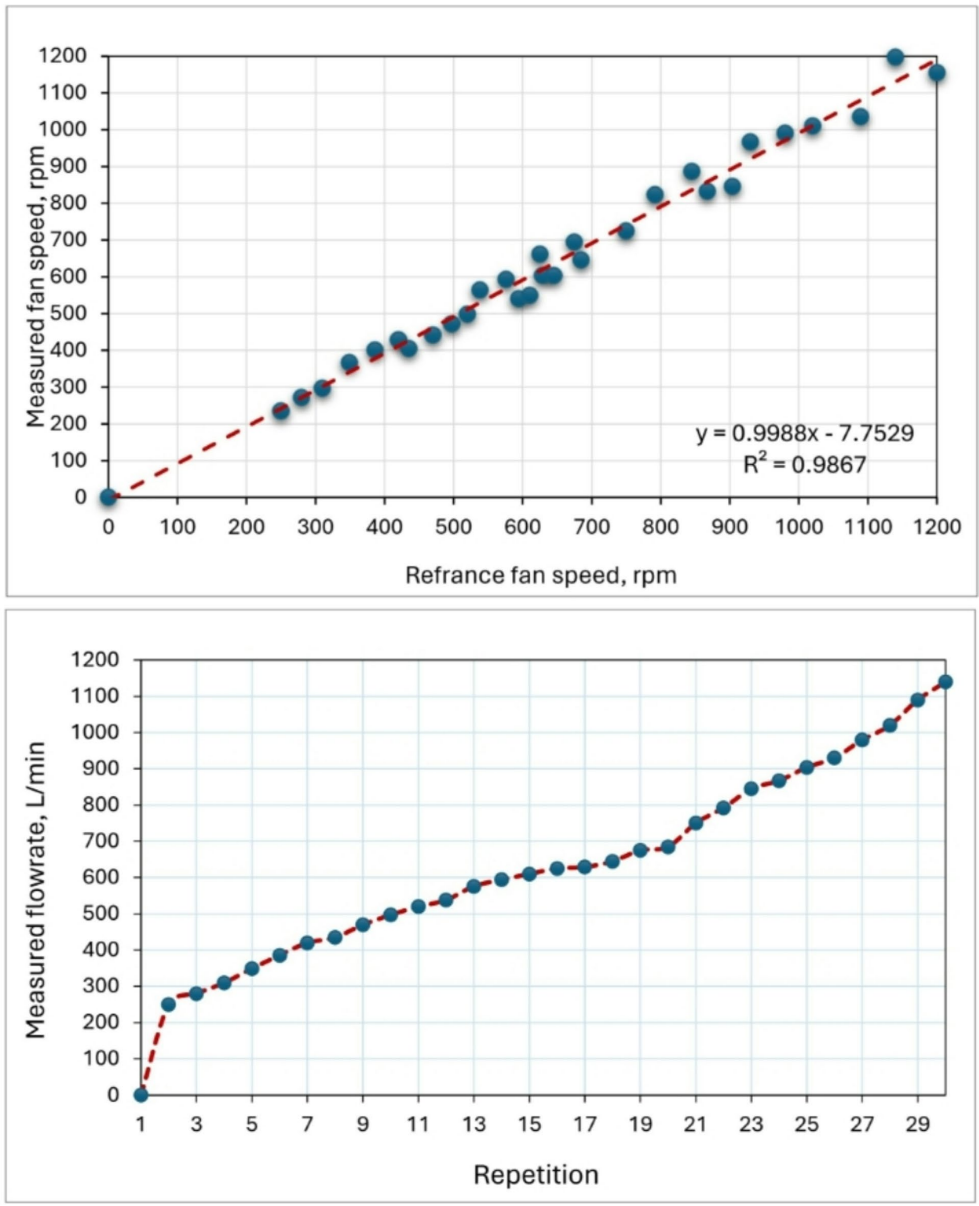
Fan speed measured by the developed measuring system.

Figure 13 illustrates fan speed measured by the developed measuring system. The reference fan speed was measured by the speed sensor (model: LM393IC). While the operated speed measured by the Uni-T Tachometer (model: UT371) in a range of 250–1200 RPM. Ideally, both values should be identical, indicating a perfect calibration. However, a slight difference exists between them, and this disparity is mathematically represented by the equation y = 0.9988x–7.7529, which demonstrates a linear relationship between the two values. The gradient of the line is 0.9988, and the y-intercept is − 7.7529, which implies that the sensor underestimates the speed by a small amount. The value of R^2^, which is 0.9867, indicates a strong correlation between the observed and operated speeds. Many previously used the speed sensor model LM393IC for measuring the speed of different devices such as, Pravinth Raja et al.^49^ used it for designing a smart steering wheel for improving driver’s safety based on IoT. Elwakeel et al.^50^ reported that the speed measurements obtained using the LM393IC sensor were validated against observations from a stepper motor integrated with a ground wheel, achieving a high R^2^ value of 0.9986.

### Ammonia (NH_3_) sensor

**Fig. 14:**
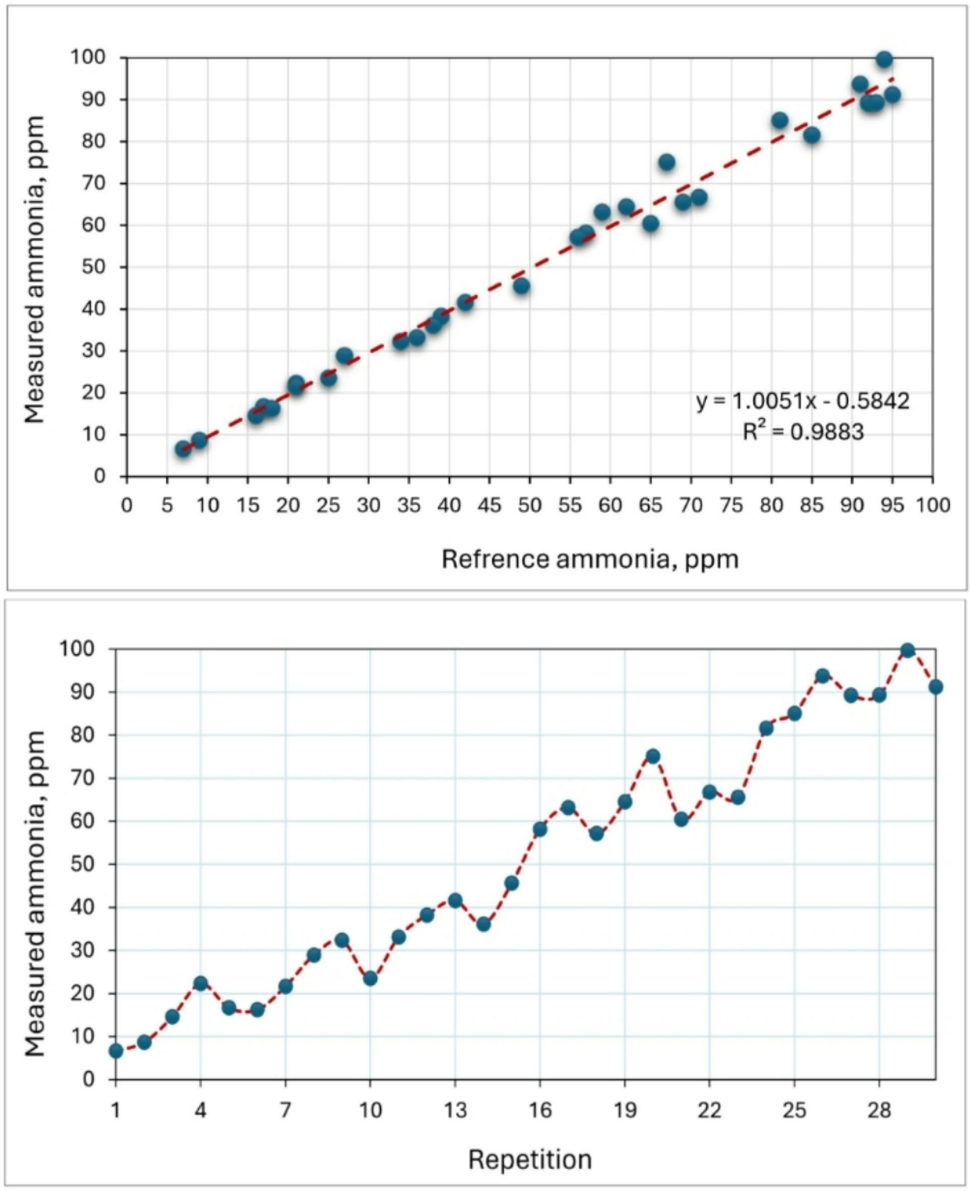
Calibration of the NH_3_ sensor by reference measuring device.

When analyzing the NH_3_ content in the air, it was seen that the sensors showed more similar values at lower amounts of NH_3_ (Fig. 14). The NH_3_ sensor (model: MQ-137) can be utilized to measure air NH_3_ levels of up to 500 ppm. The accuracy of the MQ-137 sensor in measuring NH_3_ has been confirmed through validation against a reference ammonia gas detector (model: BT-5800G) in a range of 5–95 ppm. The high R^2^ values of 0.9883 indicate a strong correlation between the detected CH_4_ values by the MQ-137 sensor and the reference device, suggesting a perfect match. The MQ-137 sensor demonstrated excellent performance over a range of CH_4_ concentrations and exhibited robust linear regressions (y = 1.0051 x) that closely aligned with the 1:1 line. The findings are in agreement with Fernando et al. (Fernando et al. 2020), who reported that the NH_3_ sensor (model: MQ-137) is capable of detecting air NH_3_ concentrations of up to roughly 60 ppm. Furthermore, they indicated that this constraint is linked to the sensor’s physical characteristics and design, as well as the impact of temperature and humidity. This issue can be resolved by incorporating these variables into the calibration curve for future iterations. Nevertheless, it is important to acknowledge that elevated levels of airborne NH_3_, above 10 ppm, can potentially jeopardize the well-being and health of both poultry and farm personnel^51,52^. The correlation coefficient for NH_3_, which is R^2^ = 0.9883, can be regarded as a strong and favorable outcome.

The MQ-137 sensor operates on a resistive principle, where the sensor resistance ($\:{R}_{s}$​) varies with the concentration of ammonia gas. Since the manufacturers provide only sensitivity curves in logarithmic scale, we experimentally derived the calibration equations by exposing MQ-137 sensor to different ammonia gas concentrations and recording the corresponding output voltage. The obtained data were fitted to empirical models that relate ammonia concentration ($\:{C}_{{NH}_{3}}$, in ppm) to the measured output voltage ($\:{V}_{out}$​), where the best-fit model was expressed as:

$$\:{C}_{{NH}_{3}}={\propto}_{2}\times\:{V}_{out}^{b2}$$

where: $\:{\propto}_{1}\:$and $\:b1$ are experimentally determined constants ($\:{\propto}_{1}$=189.4 and b1​= −1.08).

### Methane (CH_4_) sensor

The laboratory atmosphere was used to test the accuracy of measuring the CH_4_ sensor (model: MQ-4) by exposing it to various concentrations ranged between 250 and 1450 ppm. The accuracy of the MQ-4 sensor in measuring CH_4_ has been confirmed through validation against a reference methane gas detector (model: CGD02A). Prior to its use for gas measurement, this sensor, like any other, necessitates a preheating procedure to ensure reliable reading. According to the test results, the average preheating time was 75.8 s. These findings are consistent with Adhim et al.^53^ who utilized a MQ-4 sensor to detect the concentration of CH_4_ in the open air. They reported that, according to the results of laboratory tests, the average preheating time was 78.2 s.

**Fig. 15:**
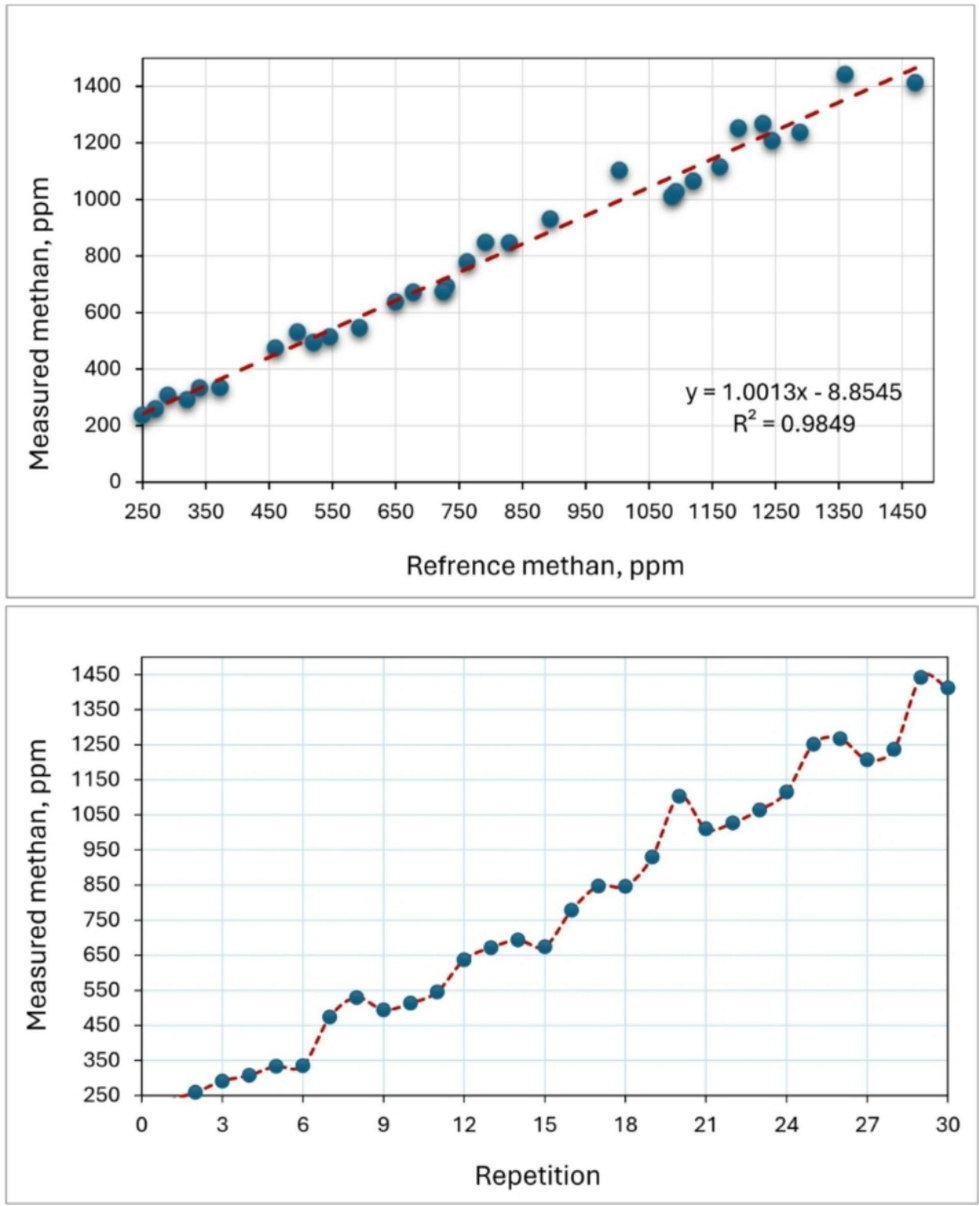
Calibration of the CH_4_ sensor by reference measuring device.

Furthermore, according to the acquired findings, the R^2^ coefficient was determined to be 0.9849 (Fig. 15). This number closely aligns with the results reported by (Ali et al. 2020). They presented a procedure that utilizes a versatile capsule for the MQ-4 sensor to accurately monitor elevated levels of CH_4_. The method involves the process of mitigating the concentration of the gas under examination by mixing it with a measured amount of air. The study compares and evaluates three different approaches in terms of their linearity and repeatability for measuring purposes. The initial approach involved doing the procedure within a sealed chamber, while the second approach involved directly introducing the gas onto the sensor in an exposed setting. Lastly, the final approach entailed directly injecting the gas into the sensor within a partially enclosed capsule. Comparisons indicate that the first technique has the highest level of repeatability, with a maximum R^2^ value of 0.8637. The second approach has superior linearity but poor reproducibility. The third strategy yields the most favorable outcomes, as evidenced by R^2^ values of 0.9973.

The MQ4 sensor operates on a resistive principle, where the sensor resistance ($\:{R}_{s}$​) varies with the concentration of methane gas. Since the manufacturers provide only sensitivity curves in logarithmic scale, we experimentally derived the calibration equations by exposing MQ4 sensor to different methane gas concentrations and recording the corresponding output voltage. The obtained data were fitted to empirical models that relate methane concentration ($\:{C}_{{CH}_{4}}$) to the measured output voltage ($\:{V}_{out}$​), where the best-fit model was expressed as:

$$\:{C}_{{CH}_{4}}={\propto\:}_{1}\times\:{V}_{out}^{b1}$$

where: $\:{\propto\:}_{1}\:$and $\:b1$ are experimentally determined constants ($\:{\propto\:}_{1}$=215.6 and b1​= −1.12).

### Cost of proposed control and monitoring system

As shown in Table 2, the total cost of the measuring system that is used to measure the concentration of CH_4_ and NH_3_ as well as the temperature and relative humidity of the atmosphere of poultry houses was only 37.5 USD. Compared to reference devices used for the same purposes, the cost of the measuring system is significantly lower. Where the cost of the temperature and humidity meter (model: UT333s) is about 17 USD, the cost of the ammonia meter (model: BT-5800G) is about 199 USD and the cost of the methane meter (model: BT-5800G) is about 105 USD. So, the total cost of the three reference devices is about 321 USD. This means that the cost of the developed AMCU represents 11.68% of the total cost of the commercial reference devices.

The tabulated data in the same table shows that the construction cost of the earlier warning system is 38.5 USD, this cost is very low compared to the many benefits of that system, such as minimizing the death rate, maximizing the production rate, and mitigating the emission of a GHG like CH_4_ and NH_3_.

**Table 2:** Cost of both measuring system and earlier waning system.

No.	Component	Cost, USD
	Measuring system	
1.	Arduino mega board	21.0
2.	CH_4_ senor	4.0
3.	NH_3_ sensor	4.0
4.	Temperature and humidity sensor	3.5
5.	Relay	5.0
Total		37.5
Earlier warning system		
6.	Speed sensor	3.5
7.	Water flowrate sensor	11.0
8.	Wi-Fi module	9.0
9.	GSM module	15.0
Total		38.5

### Comparative analysis between the developed AMCU and other available systems

Table 3 displays the comparison between the experimental findings of the present study and those of other researchers globally. Experimental investigations were conducted in the laboratory to validate the dependability of the experiments and the accuracy of the theory. The proposed system offers a notable edge over other systems described in literature as it can precisely detect sugarcane stalk nodes. This capability assists farmers in producing sugarcane seeds at a remarkably low expense compared to alternative methods. Additionally, the system boasts a swift identification process and achieves a recognition rate of up to 100%. Collaborating with manufacturers will enhance the system and facilitate mass manufacturing, hence promoting agricultural mechanization and productivity.

**Table 3:** Comparison of the current study with previous studies.

Reference	Technology	Farm type	Environmental parameters	Controller type	Earlier alarming system
^54^	Wireless Sensor Network	Poultry farm	Temperature	Arduino nano with XBee and UARTS Bee	No existing
^11^	IoT	Poultry farm	Temperature, humidity, NH_3_, luminosity	ESP-8266EXwith standard Wi-Fi	No existing
^24^	Wireless Sensor Network	Livestock Buildings	CO_2_, Temperature, and humidity	CC1110F32 integrated with CSV database	No existing
^9^	IoT	Poultry houses	Temperature, humidity, luminosity, CO_2_, wind speed	PLC; model: ECM6L45160 and ECI2626440	No existing
^22^	IoT	Poultry houses	Temperature, humidity, luminosity	Raspberry Pi with Wireless AP	No existing
^55^	IoT	Poultry farm	Temperature, humidity, water level	Arduino Uno	No existing
^56^	IoT	Animal farm	Temperature, humidity, NH_3_, CO_2_	ESP 32	No existing
^57^	IoT	Livestock farm	Temperature, humidity	FX3U-32MR PLC	No existing
^58^	IoT	Livestock farm	Temperature, luminosity	Arduino and XBee	No existing
Current study	IoT	Poultry houses	Temperature, humidity, NH_3_, and CH_4_	Arduino Mega 2650 integrated with Wi-Fi and GSM	Available

## Conclusion and future work

Although recent studies have demonstrated the benefits of smart tools in modern poultry houses, IoT-based control and monitoring systems are not yet widely adopted. Many technologies still need to be integrated into the poultry industry, which is a key focus for the third-generation model or green revolution. To address challenges in modern poultry production—such as poultry health, environmental stress, automatic monitoring, behavior, and welfare issues—and to advance the industry towards sustainability, technological innovation is crucial. This study introduces a foundational approach to remote monitoring of temperature, relative humidity, NH_3_, and CH_4_ in poultry houses using IoT and GSM modules. By meeting the practical needs of poultry production, this approach could greatly benefit future generations and is expected to drive the development of smart poultry farming. The validation results of the DHT-11 sensor showed high correlations, with R^2^ values of 0.9782 for air temperature and 0.9639 for air humidity. Additionally, the findings suggest that the NH_3_ sensor (model: MQ-137) can be utilized to measure air NH_3_ levels of up to 70 ppm. The high R^2^ values of 0.9883 indicate a strong correlation between the detected CH_4_ values by the MQ-137 sensor and the reference device, suggesting a perfect match. Furthermore, according to the acquired findings, the R^2^ coefficient of the CH_4_ sensor (model: MQ-4) was determined to be 0.9849 which indicated a prefect matching between the measured and reference data. On the other hand, the total cost of the measuring system was only 37.5 USD. That is much lower than the cost of reference devices that are used for the same purposes.

This study presents a low-cost IoT–GSM system for monitoring temperature, humidity, NH_3_, and CH_4_ in poultry houses, showing strong sensor correlations and reliable performance at only 37.5 USD. While practical limitations include dependence on GSM coverage and the need for periodic sensor calibration, the system provides an effective and affordable alternative to expensive reference devices. Theoretically, it establishes a foundation for smart poultry farming, with potential for expansion into broader environmental and welfare monitoring applications.

### Future work

The developed system will be tested by measuring ammonia and methane gas emissions in a poultry farm that does not use the developed system (traditional farm) and comparing it to another using the developed system under tropical weather conditions (Aswan, Egypt). The emissions in the two barns will be compared to judge the system’s effectiveness during the whole season.

## Data Availability

All data are presented within the article.
